# Supplementary material for: Cu14H12(PtBu3)6Cl2—The Expanse of Stryker’s Reagent
Source: Molecules. 2025 Dec 15;30(24):4779. doi: 10.3390/molecules30244779 (PMC12735477; doi:10.3390/molecules30244779)
Supplement: Supplementary file 1 [file molecules-30-04779-s001.zip › molecules-4028172-supplementary.pdf]

Supporting Information

$Cu_{14}H_{12}(P^tBu_3)_6Cl_2$—The Expanse of Stryker's Reagent

Markus Strienz †, Roman Kimmich †, Alexander Conzelmann and Andreas Schnepf *

Institut für Anorganische Chemie, Universität Tübingen, Auf der Morgenstelle 18, 72076 Tübingen, Germany

* Correspondence: andreas.schnepf@uni-tuebingen.de

† These authors contributed equally to this work.

Table of Contents

1 NMR-data 3

1.1 NMR-spectra of $Cu_{14}(P^{t}Bu_3)_6Cl_2H_{12}$ (**2**) 3

1.2 NMR-spectra of the reaction of **2** with Cyclohex-2-en-1-one 5

2 Crystallographic data 6

3 UV/Vis-data 7

4 DLS-measurements 8

5 ESI-MS-measurements 9

6 Theoretical calculations 12

7 Machine Learning Architecture and Hyperparameters 16

1 NMR-data

1.1 NMR-spectra of $Cu_{14}(P^{t}Bu_3)_6Cl_2H_{12}$ (**2**)

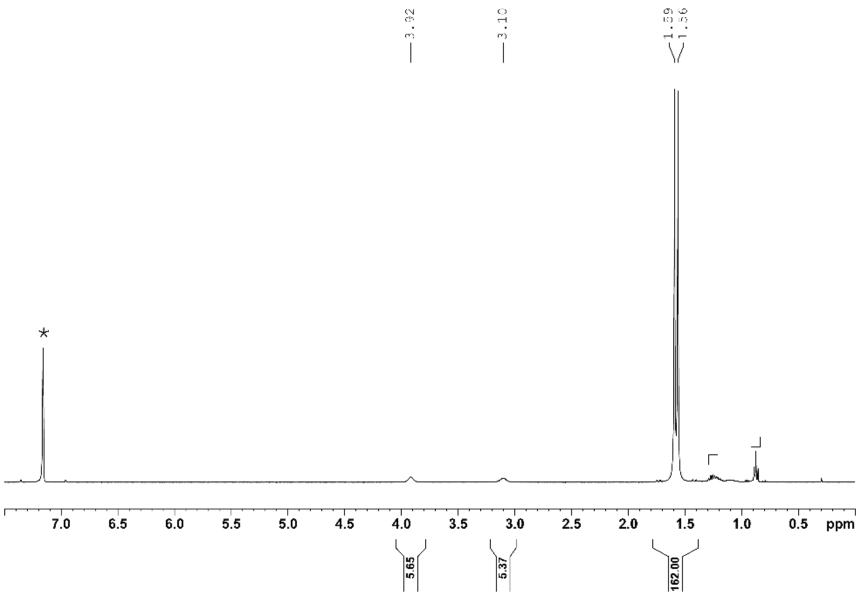

Figure S1: ^{1}H-NMR spectrum of **2** (400.1 MHz, C_6D_6). * = solvent residual signal, □ = co-crystallized *n*-pentane.

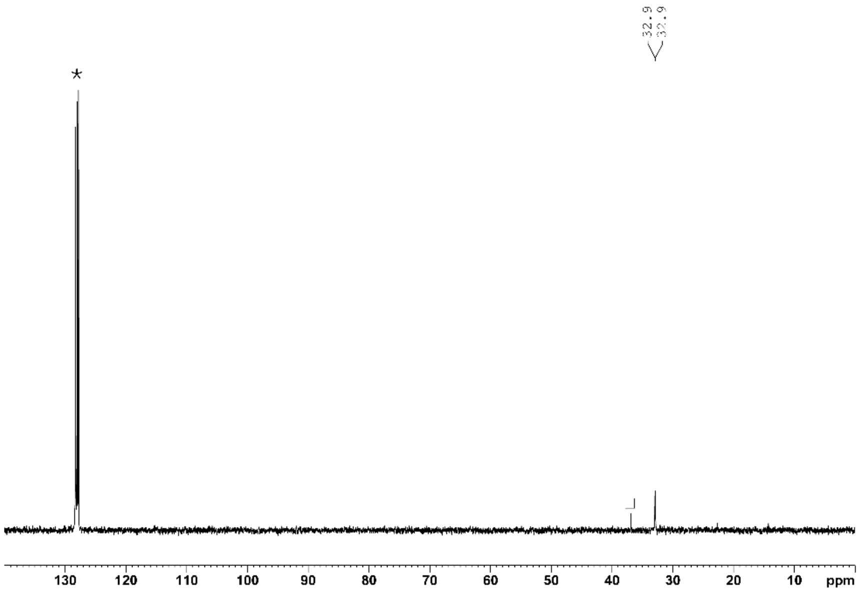

Figure S2: ^{13}C-udeft-NMR spectrum of **2** (100.6 MHz, C_6D_6). * = solvent residual signal, □ = co-crystallized *n*-pentane.

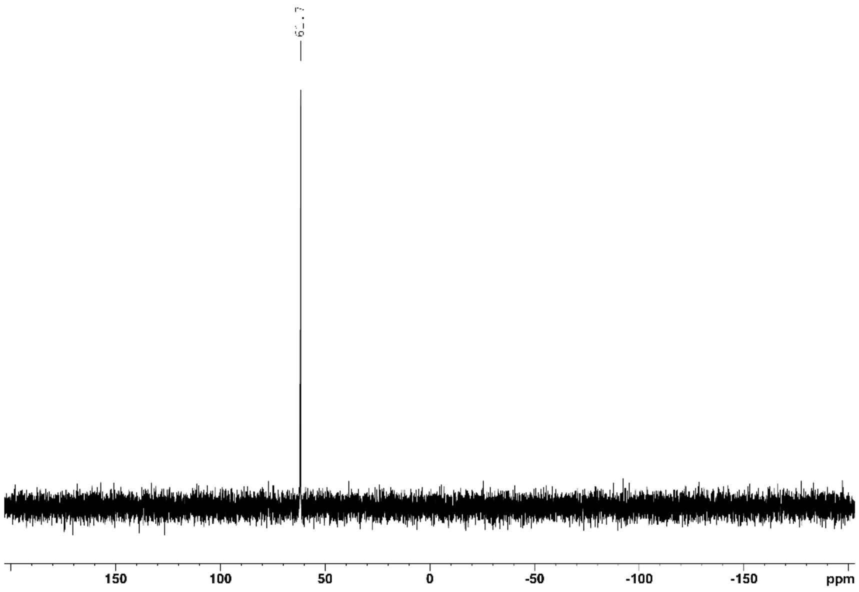

Figure S3: $^{31}P\{^{1}H\}$-NMR spectrum of **2** (162.0 MHz, C_6D_6).

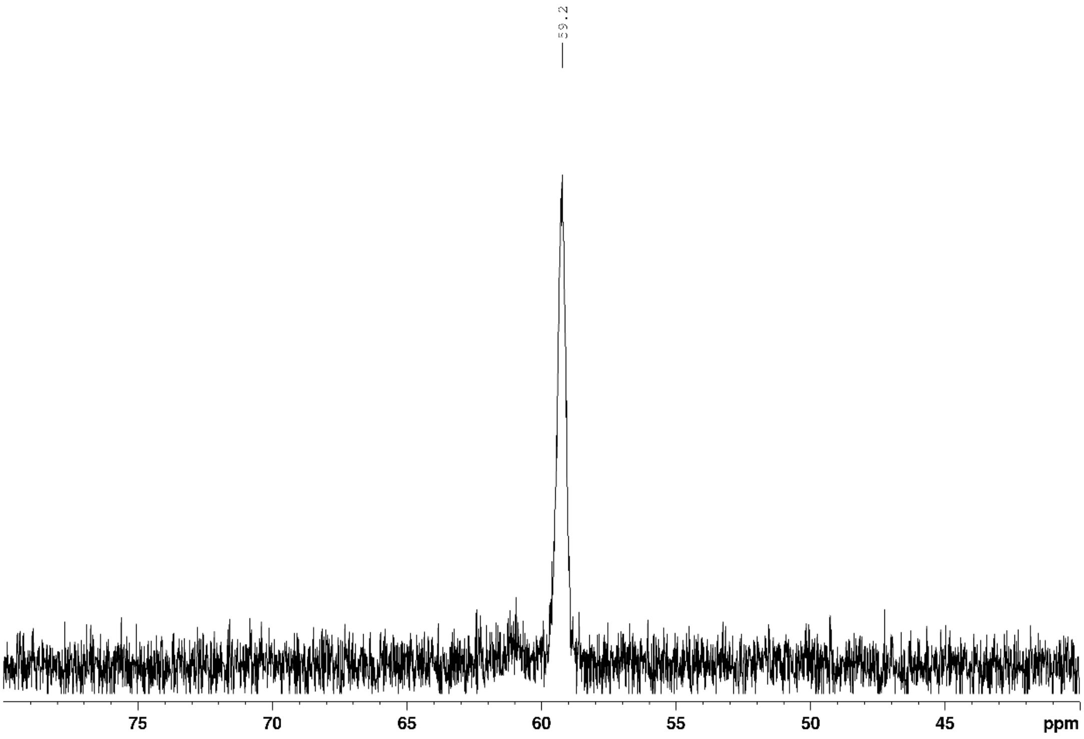

Figure S4: ^{31}P-NMR spectrum of **2** (242.9 MHz, thf-d_8).

1.2 NMR-spectra of the reaction of **2** with Cyclohex-2-en-1-one

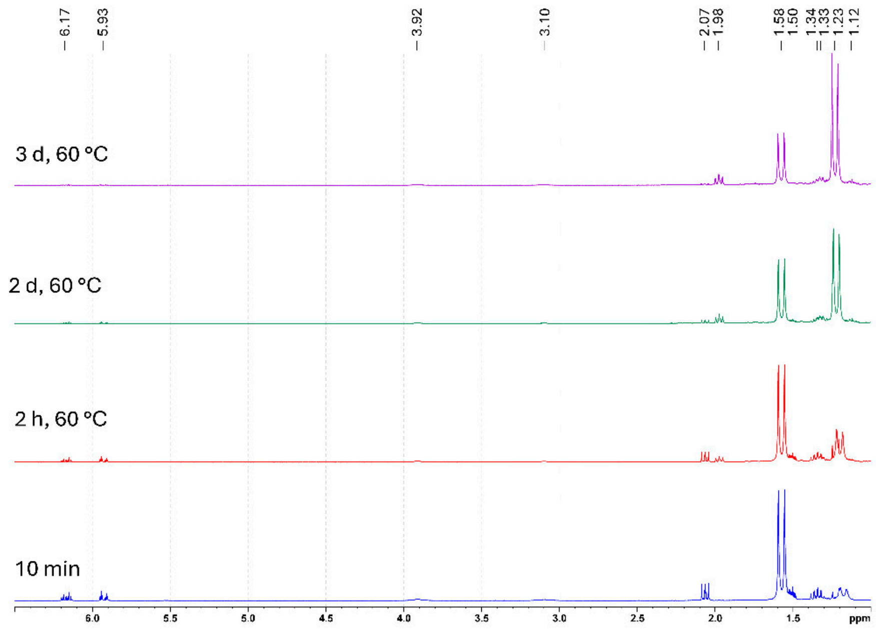

Figure S5: ^{1}H-NMR spectra (400.1 MHz, C_6D_6) of **2** with cyclohex-2-en-1-one over a period of three days.

2 Crystallographic data

Compound	**2**
Empirical formula	$C_{82}H_{198}Cl_2Cu_{14}P_6$
Formula weight	2330.67
Temperature/K	100.00
Crystal system	triclinic
Space group	P-1
a/Å	13.9699(7)
b/Å	14.9337(7)
c/Å	15.5710(7)
α/°	61.4420(10)
β/°	78.328(2)
γ/°	68.932(2)
Volume/Å^3	2660.7(2)
Z	1
ρ_{calc}/g/cm^3	1.455
μ/mm^{-1}	2.909
F(000)	1220.0
Crystal size/mm^3	0.296 × 0.269 × 0.165
Radiation	MoKα (λ = 0.71073)
2Θ range for data collection/°	4.238 to 61.128
Index ranges	-19 ≤ h ≤ 19, -21 ≤ k ≤ 21, -22 ≤ l ≤ 22
Reflections collected	91203
Independent reflections	16225 [R_{int} = 0.0425, R_{sigma} = 0.0290]
Data/restraints/parameters	16225/66/724
Goodness-of-fit on F^2	1.040
Final R indexes [I>=2σ (I)]	R_1 = 0.0321, wR_2 = 0.0845
Final R indexes [all data]	R_1 = 0.0418, wR_2 = 0.0907
Largest diff. peak/hole / e Å^{-3}	1.17/-0.70
CCDC	2504326

3 UV/Vis-data

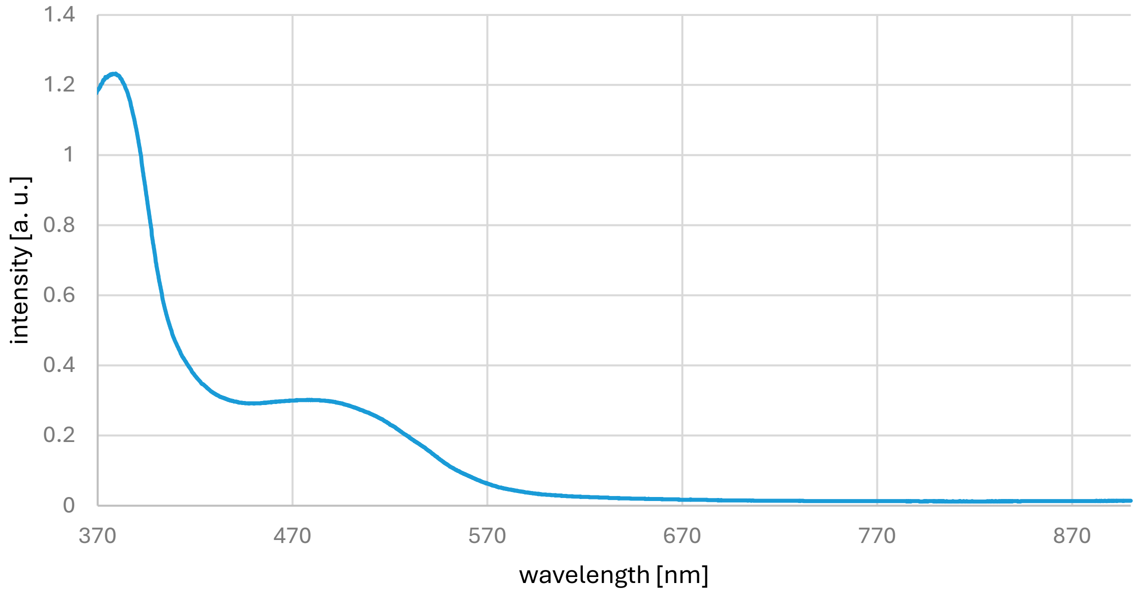

Figure S6: Absorption spectrum of **2** in benzene at room temperature.

4 DLS-measurements

**Sample Name:**	Cu14+Furmat 3		
**SOP Name:**	mansettings.nano		
**File Name:**	Cu14+Furmat.dts	**Dispersant Name:**	Benzol
**Record Number:**	3	**Dispersant RI:**	1.496
**Material RI:**	0.14	**Viscosity (cP):**	0.6030
**Material Absorbtion:**	0.000	**Measurement Date and Time:**	Dienstag, 14. Oktober 2025 15:37:19

**Temperature (°C):**	25.1	**Duration Used (s):**	70
**Count Rate (kcps):**	220.5	**Measurement Position (mm):**	0.65
**Cell Description:**	Glass cuvette with round aperture	**Attenuator:**	10

			Size (d.nm):	% Number:	St Dev (d.nm):
**Z-Average (d.nm):**	75.62	**Peak 1:**	9.084	100.0	2.356
**PdI:**	0.377	**Peak 2:**	0.000	0.0	0.000
**Intercept:**	0.399	**Peak 3:**	0.000	0.0	0.000

Result quality : **Refer to quality report**

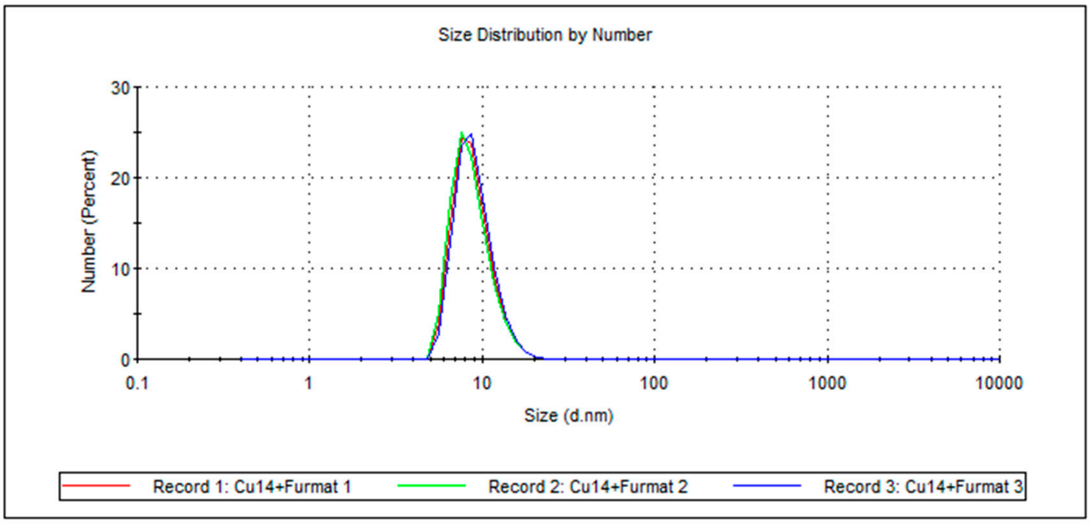

5 ESI-MS-measurements

Mass Spectrum SmartFormula Report

**Analysis Info**		Acquisition Date	14.08.2025 10:12:12
Analysis Name	M:\Test_nur fuer MS-Abt\Schnepf\Schnepf_Kimmich_Strienz_Cu14-Cluster_000001.d		
Method	tune_100-3000_pos_neu.m	Operator	BDAL@DE
Sample Name	Schnepf_Kimmich_Strienz_Cu14-Cluster_	Instrument	maXis 288882.21253
Comment	ACN geloest; ACN verduennt		

Acquisition Parameter

Source Type	ESI	Ion Polarity	Positive	Set Nebulizer	0.3 Bar
Focus	Active	Set Capillary	3800 V	Set Dry Heater	200 °C
Scan Begin	100 m/z	Set End Plate Offset	-500 V	Set Dry Gas	4.0 l/min
Scan End	3000 m/z	Set Charging Voltage	0 V	Set Divert Valve	Waste
		Set Corona	0 nA	Set APCI Heater	0 °C

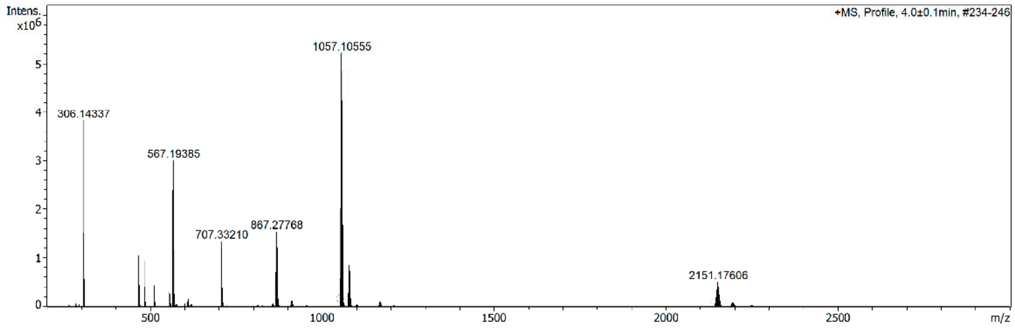

	Meas. m/z	#	Ion Formula	m/z	err [ppm]	mSigma	#mSigma	Score	rdb (neutral)	e— Conf	N-Rule
[M-2Cl]²⁺	1053.10657	1	C72H174Cu14P6	1053.10870	2.02	39.3	1	100.00	-18.0	even	ok
[M-Cl]⁺	2141.18322	1	C72H174ClCu14P6	2141.18679	1.67	14.5	1	100.00	-18.5	even	ok

Schnepf_Kimmich_Strienz_Cu14-Cluster_000001.d
Bruker Compass DataAnalysis 6.1 printed: 14.08.2025 15:25:36 by: Norbert Page 1 of 1

Display Report

**Analysis Info**		Acquisition Date	14.08.2025 10:12:12
Analysis Name	M:\Test_nur fuer MS-Abt\Schnepf\Schnepf_Kimmich_Strienz_Cu14-Cluster_000001.d		
Method	tune_100-3000_pos_neu.m	Operator	BDAL@DE
Sample Name	Schnepf_Kimmich_Strienz_Cu14-Cluster_	Instrument	maXis 288882.21253
Comment	ACN geloest; ACN verduennt		

Acquisition Parameter

Source Type	ESI	Ion Polarity	Positive	Set Nebulizer	0.3 Bar
Focus	Active	Set Capillary	3800 V	Set Dry Heater	200 °C
Scan Begin	100 m/z	Set End Plate Offset	-500 V	Set Dry Gas	4.0 l/min
Scan End	3000 m/z	Set Charging Voltage	0 V	Set Divert Valve	Waste
		Set Corona	0 nA	Set APCI Heater	0 °C

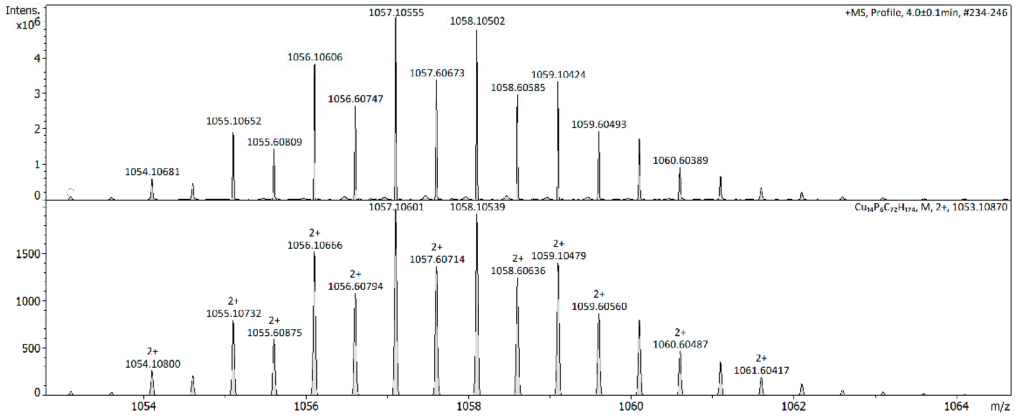

Schnepf_Kimmich_Strienz_Cu14-Cluster_000001.d
Bruker Compass DataAnalysis 6.1 printed: 14.08.2025 15:30:07 by: Norbert Page 1 of 1

Display Report

Analysis Info

Analysis Name	M:\Test_nur fuer MS-Abt\Schnepf\Schnepf_Kimmich_Strienz_Cu14-Cluster_000001.d	Acquisition Date	14.08.2025 10:12:12
Method	tune_100-3000_pos_neu.m	Operator	BDAL@DE
Sample Name	Schnepf_Kimmich_Strienz_Cu14-Cluster_	Instrument	maXis 288882.21253
Comment	ACN geloest; ACN verduennt		

Acquisition Parameter

Source Type	ESI	Ion Polarity	Positive	Set Nebulizer	0.3 Bar
Focus	Active	Set Capillary	3800 V	Set Dry Heater	200 °C
Scan Begin	100 m/z	Set End Plate Offset	-500 V	Set Dry Gas	4.0 l/min
Scan End	3000 m/z	Set Charging Voltage	0 V	Set Divert Valve	Waste
		Set Corona	0 nA	Set APCI Heater	0 °C

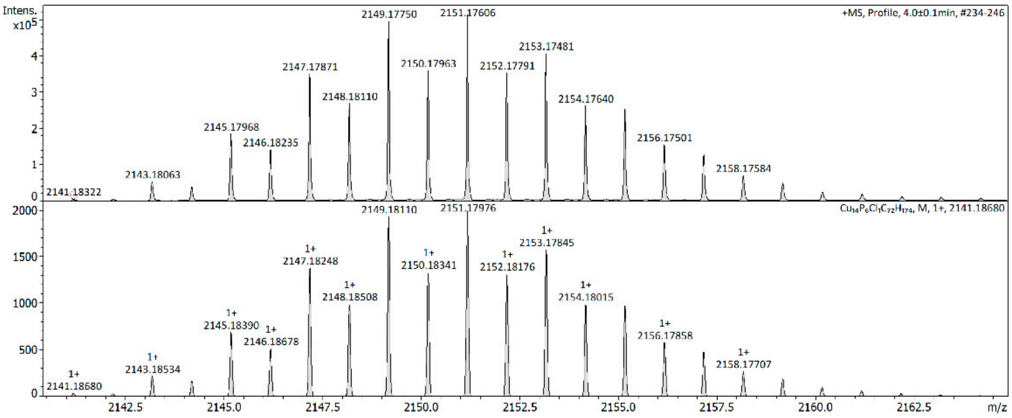

Schnepf_Kimmich_Strienz_Cu14-Cluster_000001.d
Bruker Compass DataAnalysis 6.1 printed: 14.08.2025 15:27:25 by: Norbert Page 1 of 1

Display Report

Analysis Info

Analysis Name	M:\Test_nur fuer MS-Abt\Schnepf\Schnepf_Kimmich_Strienz_Cu14-Cluster_000001.d	Acquisition Date	14.08.2025 10:12:12
Method	tune_100-3000_pos_neu.m	Operator	BDAL@DE
Sample Name	Schnepf_Kimmich_Strienz_Cu14-Cluster_	Instrument	maXis 288882.21253
Comment	ACN geloest; ACN verduennt		

Acquisition Parameter

Source Type	ESI	Ion Polarity	Positive	Set Nebulizer	0.3 Bar
Focus	Active	Set Capillary	3800 V	Set Dry Heater	200 °C
Scan Begin	100 m/z	Set End Plate Offset	-500 V	Set Dry Gas	4.0 l/min
Scan End	3000 m/z	Set Charging Voltage	0 V	Set Divert Valve	Waste
		Set Corona	0 nA	Set APCI Heater	0 °C

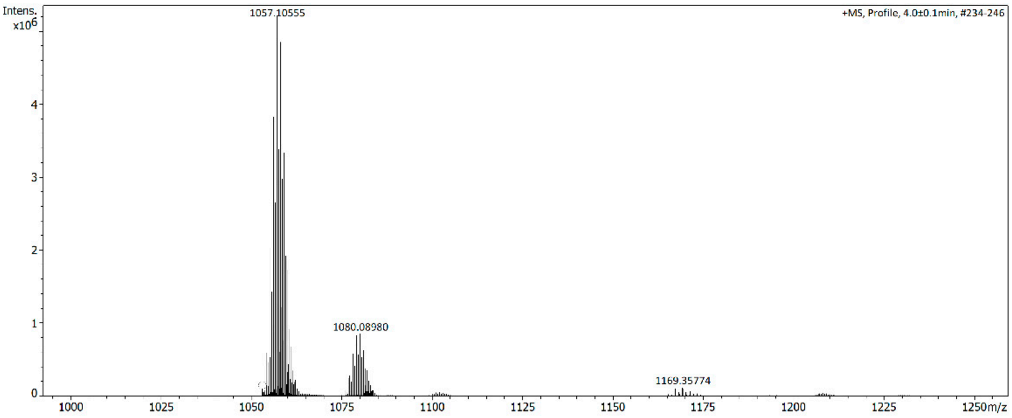

Schnepf_Kimmich_Strienz_Cu14-Cluster_000001.d
Bruker Compass DataAnalysis 6.1 printed: 14.08.2025 15:31:09 by: Norbert Page 1 of 1

Display Report

Analysis Info

Analysis Name	M:\Test_nur fuer MS-Abt\Schnepf\Schnepf_Kimmich_Strienz_Cu14-Cluster_000001.d	Acquisition Date	14.08.2025 10:12:12
Method	tune_100-3000_pos_neu.m	Operator	BDAL@DE
Sample Name	Schnepf_Kimmich_Strienz_Cu14-Cluster_	Instrument	maXis 288882.21253
Comment	ACN geloest; ACN verduennt		

Acquisition Parameter

Source Type	ESI	Ion Polarity	Positive	Set Nebulizer	0.3 Bar		
Focus	Active	Set Capillary	3800 V	Set Dry Heater	200 °C		
Scan Begin	100 m/z	Set End Plate Offset	-500 V	Set Dry Gas	4.0 l/min		
Scan End	3000 m/z	Set Charging Voltage	0 V	Set Divert Valve	Waste		
		Set Corona	0 nA	Set APCI Heater	0 °C		

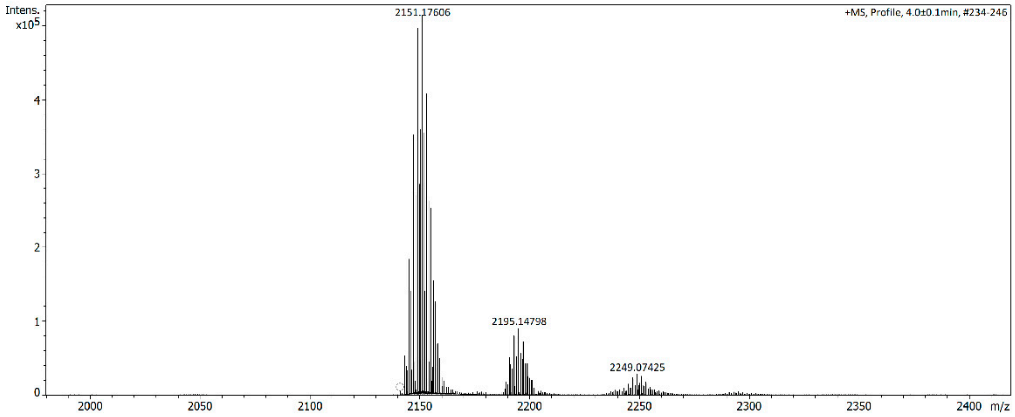

Schnepf_Kimmich_Strienz_Cu14-Cluster_000001.d
Bruker Compass DataAnalysis 6.1 printed: 14.08.2025 15:31:32 by: Norbert Page 1 of 1

6 Theoretical calculations

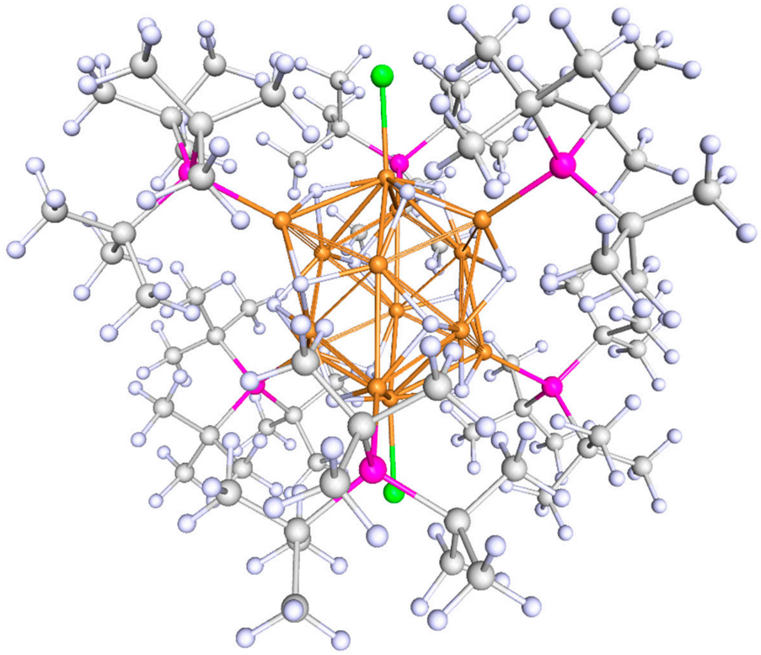

Figure S7: Minimum structure of **2**.

Level of theory: BP86-D3BJ/def2-SV(P)//PBE0-D3BJ/def2-TZVP

Total energy: -28778.69085138031 H

HOMO-LUMO gap: 3.575 eV

Table S1: Atom coordinates of the calculated minimum structure of **2**

1	Cu	11.1504	10.4881	4.8069	135	H	7.1506	10.4301	8.5843
2	Cu	12.289	12.2817	6.1543	136	H	5.4853	10.6655	9.126
3	Cu	9.6158	10.2602	6.7859	137	H	15.4871	11.7834	-0.2633
4	Cu	12.7993	8.4799	5.3875	138	H	14.396	10.5537	-0.9009
5	Cu	13.6019	10.8638	4.4857	139	H	15.7887	10.072	0.0562
6	Cu	10.1128	8.3013	5.2596	140	H	16.3194	10.2645	5.082
7	Cu	9.86	12.5151	5.7758	141	H	17.3527	11.6589	4.7522
8	Cu	13.8541	10.4382	6.8825	142	H	17.9845	10.0294	4.5396
9	Cu	10.6707	12.2184	8.2808	143	H	6.8797	6.8881	6.4668
10	Cu	13.357	12.397	8.4089	144	H	7.3455	8.5315	6.0443
11	Cu	11.181	8.4166	7.514	145	H	5.6676	8.0038	5.8431
12	Cu	9.8683	9.8344	9.1827	146	H	13.901	7.7345	12.2042

13	Cu	13.61	8.1832	7.8925	147	H	13.1603	6.5162	11.1694
14	Cl	13.804	6.8524	4.1094	148	H	13.322	8.1719	10.5906
15	P	14.9654	11.4424	2.8028	149	H	12.9245	9.231	0.5614
16	P	8.819	6.8833	4.0976	150	H	12.7758	9.5703	2.2899
17	P	8.4017	13.9805	4.8953	151	H	14.1874	8.7347	1.6887
18	C	9.4221	8.4206	1.8454	152	H	7.5153	5.7386	12.0862
19	C	9.6568	13.3104	2.4977	153	H	6.7497	7.272	12.5002
20	Cu	12.3196	10.2102	8.8616	154	H	8.3971	6.9302	13.0317
21	Cl	9.6662	13.8469	9.5579	155	H	6.4489	11.6583	5.0625
22	P	14.651	13.8151	9.5707	156	H	7.3025	11.8495	6.5942
23	P	8.5045	9.2563	10.8655	157	H	5.5589	12.1232	6.512
24	C	6.7131	7.6905	5.7539	158	H	18.6479	10.4986	2.1931
25	P	15.0689	6.7182	8.7725	159	H	17.4924	11.0949	1.0046
26	C	16.6474	10.5793	2.9576	160	H	18.0394	12.1432	2.3173
27	C	15.2334	13.3239	2.7725	161	H	7.6626	4.011	3.6517
28	C	14.1175	10.8974	1.1971	162	H	8.9289	3.0467	4.4107
29	C	16.4565	9.0879	2.6728	163	H	9.283	3.955	2.9491
30	C	13.9095	14.0188	3.1115	164	H	8.4857	8.972	1.8369
31	C	13.4818	9.5263	1.457	165	H	9.8327	8.4492	0.8311
32	C	9.1614	5.1464	4.7756	166	H	10.1221	8.9381	2.5039
33	C	9.2721	6.95	2.2562	167	H	15.324	15.4906	12.1145
34	C	6.9722	7.2839	4.299	168	H	14.7666	14.3941	13.3755
35	C	10.6651	5.0504	5.0566	169	H	16.1489	13.9518	12.3834
36	C	8.9716	15.7759	5.1102	170	H	8.702	6.3073	0.2924
37	C	8.2504	13.5949	3.039	171	H	8.1458	5.2094	1.5527
38	C	6.7129	13.7516	5.7268	172	H	7.32	6.748	1.2855
39	C	9.4991	15.9497	6.5381	173	H	17.8458	5.3157	8.9076
40	C	6.5016	12.2528	5.9707	174	H	18.8514	6.5177	8.1028
41	C	16.7568	13.0057	7.9155	175	H	18.1191	6.8908	9.6575
42	C	14.3099	15.5516	8.891	176	H	7.0974	16.8048	5.5465
43	C	14.1971	13.7501	11.412	177	H	7.4978	16.7736	3.8261
44	C	16.4976	13.4135	9.3701	178	H	8.3826	17.8291	4.9266
45	C	12.8064	15.6483	8.6093	179	H	17.3865	8.5754	2.9391
46	C	14.046	12.2799	11.8238	180	H	15.6576	8.6416	3.2705
47	C	6.822	10.1181	10.7091	181	H	16.2655	8.8775	1.6216
48	C	8.2376	7.3747	10.8979	182	H	17.9111	8.5789	7.1586

49	C	9.3509	9.8037	12.4712	183	H	17.0206	9.0418	8.6084
50	C	7.0121	11.6099	10.9923	184	H	16.1674	8.8518	7.0763
51	C	9.5621	6.6801	10.5605	185	H	15.3051	5.1077	11.5189
52	C	9.9857	11.175	12.2103	186	H	16.8908	5.7875	11.1432
53	C	14.5005	4.9225	8.5558	187	H	15.965	6.3982	12.5139
54	C	15.219	7.1023	10.6292	188	H	10.2111	7.8745	13.0927
55	C	16.7578	6.9491	7.9419	189	H	11.1131	9.3071	13.5732
56	C	13.9736	4.7495	7.1276	190	H	11.1772	8.7634	11.8967
57	C	13.8121	7.3853	11.1701	191	H	17.2125	13.4085	3.7245
58	C	16.9684	8.4482	7.6996	192	H	15.8548	13.2751	4.8601
59	C	17.0947	10.6562	4.4222	193	H	16.1678	14.794	4.0174
60	C	17.7571	11.1218	2.0614	194	H	9.2797	11.9657	11.9773
61	C	15.7773	13.8875	1.4626	195	H	10.5422	11.4716	13.1061
62	C	16.1817	13.7046	3.9106	196	H	10.6924	11.1306	11.378
63	C	15.0111	10.8289	-0.0375	197	H	10.3094	14.179	2.4975
64	C	12.946	11.8369	0.9059	198	H	10.1466	12.5239	3.0776
65	C	8.4875	5.0015	6.1418	199	H	9.5672	12.9606	1.464
66	C	8.7272	3.9862	3.8853	200	H	7.6172	7.4207	8.8099
67	C	10.6621	6.3354	2.0775	201	H	6.259	7.2879	9.945
68	C	8.295	6.2607	1.3079	202	H	7.3046	5.9026	9.6542
69	C	6.6446	8.5288	3.4747	203	H	6.7382	15.4717	7.089
70	C	6.0106	6.1653	3.9074	204	H	5.8543	14.0637	7.6569
71	C	10.178	16.0124	4.1994	205	H	7.617	14.0592	7.7072
72	C	7.9169	16.8427	4.8295	206	H	13.2582	12.8278	0.5783
73	C	7.585	14.6674	2.1821	207	H	12.2924	11.9384	1.7742
74	C	7.4887	12.2801	2.868	208	H	12.3548	11.3964	0.097
75	C	6.7534	14.3832	7.1201	209	H	12.529	5.4516	9.3235
76	C	5.5216	14.3185	4.9599	210	H	13.5547	4.634	10.5223
77	C	14.9846	15.6948	7.525	211	H	12.8499	3.7221	9.1932
78	C	14.7445	16.7123	9.7804	212	H	18.487	14.1638	9.6668
79	C	12.8073	14.3656	11.5896	213	H	17.369	15.4017	9.111
80	C	15.1742	14.4396	12.3601	214	H	17.3206	14.8614	10.7901
81	C	16.8246	12.169	10.1953	215	H	7.8111	12.056	10.3944
82	C	17.4597	14.532	9.7609	216	H	6.082	12.1217	10.725
83	C	6.3752	10.0391	9.2444	217	H	7.2026	11.8216	12.0433
84	C	5.7124	9.576	11.6056	218	H	14.0893	15.0983	3.1525

85	C	7.6932	6.8125	12.2082	219	H	13.1208	13.8402	2.3862
86	C	7.2901	6.9921	9.7598	220	H	13.54	13.6985	4.0876
87	C	8.4563	9.8729	13.705	221	H	5.6431	8.8712	3.7537
88	C	10.5228	8.8653	12.7643	222	H	6.6386	8.3421	2.4024
89	C	13.2939	4.6842	9.466	223	H	7.3424	9.3402	3.689
90	C	15.5559	3.8563	8.8359	224	H	17.6171	6.6395	6.0117
91	C	15.8849	6.0297	11.4855	225	H	15.8544	6.6428	5.961
92	C	15.9797	8.4176	10.8017	226	H	16.7341	5.2304	6.5779
93	C	16.7181	6.3189	6.5479	227	H	14.6169	16.6171	7.0646
94	C	17.9492	6.3822	8.7086	228	H	16.0696	15.7707	7.5839
95	H	9.5017	10.8718	5.202	229	H	14.7205	14.8678	6.8624
96	H	9.588	8.5184	6.927	230	H	9.9517	16.9443	6.6123
97	H	11.3348	8.8774	9.1929	231	H	10.2609	15.2086	6.777
98	H	13.9682	9.8266	8.4665	232	H	8.7376	15.8755	7.307
99	H	13.8819	12.18	6.7414	233	H	15.809	16.6871	10.0145
100	H	12.1352	11.8208	4.4754	234	H	14.1883	16.7447	10.7164
101	H	8.856	4.0791	6.6012	235	H	14.5436	17.6515	9.2542
102	H	7.4025	4.9249	6.0834	236	H	6.1497	5.8368	2.878
103	H	8.7513	5.8281	6.805	237	H	6.1017	5.2951	4.5566
104	H	12.4792	14.1619	12.6138	238	H	4.9831	6.533	4.0019
105	H	12.8031	15.4468	11.4612	239	H	5.9768	9.6043	12.6624
106	H	12.062	13.9322	10.9181	240	H	5.4309	8.5541	11.3509
107	H	5.351	13.8091	4.0116	241	H	4.8213	10.1984	11.4729
108	H	4.6195	14.1841	5.5661	242	H	13.2112	5.4902	6.8892
109	H	5.6256	15.3847	4.7599	243	H	13.5219	3.7546	7.0523
110	H	9.0704	10.1494	14.5686	244	H	14.7353	4.8252	6.359
111	H	7.9807	8.9183	13.9315	245	H	17.0386	8.3378	10.5626
112	H	7.6782	10.6291	13.6098	246	H	15.5351	9.2061	10.1926
113	H	6.4299	12.3608	3.1075	247	H	15.9018	8.7227	11.85
114	H	7.5658	11.9741	1.8198	248	H	16.1266	11.3578	9.9816
115	H	7.933	11.4918	3.4775	249	H	17.8261	11.8261	9.9167
116	H	10.8678	4.0762	5.5142	250	H	16.8306	12.3566	11.2675
117	H	11.3012	5.1473	4.1825	251	H	12.4895	14.8762	7.9041
118	H	10.9818	5.822	5.7624	252	H	12.1699	15.5525	9.4831
119	H	9.9169	16.0618	3.1432	253	H	12.6045	16.6222	8.1507
120	H	10.9423	15.2445	4.3423	254	H	9.932	6.9995	9.5843

121	H	10.623	16.9743	4.4712	255	H	10.3502	6.8599	11.286
122	H	13.6349	12.2524	12.838	256	H	9.3828	5.6005	10.5206
123	H	14.9821	11.7279	11.8332	257	H	16.7204	13.4279	1.1695
124	H	13.346	11.7623	11.1655	258	H	15.9559	14.9611	1.5859
125	H	16.1242	12.1648	7.6257	259	H	15.0729	13.7711	0.6394
126	H	16.5906	13.8077	7.202	260	H	16.3756	3.8954	8.1191
127	H	17.8022	12.692	7.8267	261	H	15.091	2.8696	8.7378
128	H	8.1655	15.5888	2.1477	262	H	15.9748	3.9249	9.8395
129	H	6.5795	14.9107	2.5246	263	H	12.0159	11.7933	9.3182
130	H	7.5041	14.2981	1.1541	264	H	11.0544	13.1654	6.8351
131	H	10.9897	6.5401	1.0533	265	H	9.2957	11.1263	8.1772
132	H	10.667	5.2541	2.205	266	H	14.1743	9.572	5.4914
133	H	11.4074	6.7687	2.7491	267	H	11.4539	8.9049	4.3503
134	H	6.1175	9.0359	8.9158	268	H	12.4156	7.5329	6.8331

7 Machine Learning Architecture and Hyperparameters

We use 4 convolutional layers with input channel sizes [7, 16, 32, 64] and output channel sizes [16, 32, 64, 128]. Each convolutional layer has kernel size 3, uses padding 1 and is followed by a ReLU activation function and a 2x2x2 max pooling layer. After the convolutional layers, the outputs are flattened, and fed into a linear classification layer with 2 outputs, the first one encoding the probability of the patch not containing a hydride, the second one encoding the probability of the patch containing a hydride. To determine which patches contain a hydride, we choose the ones where the second output has a higher value than the first one. During training, we use the focal loss with gamma=2 and alpha=[1.0, 1.0].

As the predicted coordinate we used the centre of the bounding box. As the bounding box is 2.65 Angstrom wide in each direction, each prediction has an uncertainty of 2.29 Angstrom (which is the maximum distance an atom can have to the centre of the bounding box).

For training hyperparameters, we use a batch size of 32 and train for 20 epochs. We use the AdamW optimizer with a weight decay of 0.01 and a learning rate of 0.00005 with a cosine learning rate scheduler. We add dropout to the final linear layer with a strength of 0.05.

We include the prediction results below, all measurements are in Ångström.

Table S2: Predicted hydride positions by CNN and their closest hydride position of the minimum structure

Predicted	x	y	z	Closest Match	x.1	y.1	z.1	Distance
1	10.12	10.12	10.12	1	11.33	8.87	9.19	3.88
2	10.12	7.46	7.46	2	9.58	8.51	6.92	1.69

3	10.12	10.12	7.46	3	9.29	11.12	8.17	2.20
4	10.12	7.46	4.79	4	11.45	8.90	4.35	4.06
5	10.12	12.78	4.79	5	9.50	10.87	5.20	4.20
6	12.78	12.78	4.79	6	12.13	11.82	4.47	1.45
7	12.78	10.12	7.46	7	13.96	9.82	8.46	2.50
8	12.78	10.12	4.79	8	14.17	9.57	5.49	2.72
9	10.12	12.78	7.46	9	11.05	13.16	6.83	1.41
10	12.78	7.46	7.46	10	12.41	7.53	6.83	0.53
11	10.12	10.12	4.79	11	9.50	10.87	5.20	1.11
12	12.78	12.78	7.46	12	13.88	12.18	6.74	2.09
